# Supplementary material for: In Utero Exposure to Hormonal Contraception and Mortality in Offspring with and without Cancer: A Nationwide Cohort Study
Source: Cancers (Basel). 2023 Jun 13;15(12):3163. doi: 10.3390/cancers15123163 (PMC10296431; doi:10.3390/cancers15123163)
Supplement: Supplementary file 1 [file cancers-15-03163-s001.zip › cancers-2425457-supplementary.pdf]

Supplementary Materials: In Utero Exposure to Hormonal Contraception and Mortality in Offspring with and without Cancer: A Nationwide Cohort Study

Lina Steinrud Mørch [1,*], Mads Gamborg [1], Caroline Hallas Hemmingsen [2], Charlotte Wessel Skovlund [1], Susanne Krüger Kjær [2] and Marie Hargreave [2]

[1] Cancer Surveillance and Pharmacoepidemiology, Danish Cancer Society Research Center, Strandboulevarden 49, 2100 Copenhagen, Denmark; maga@cancer.dk (M.G.); cws@cancer.dk (C.W.S.)

[2] Virus, Lifestyle, and Genes, Danish Cancer Society Research Center, Strandboulevarden 49, 2100 Copenhagen, Denmark; cahe@cancer.dk (C.H.H.); susanne@cancer.dk (S.K.K.); mariehar@cancer.dk (M.H.)

* Correspondence: morch@cancer.dk; Tel.: +45-21203670

.

Table of Contents

Table S1. Mortality in children who develop any type of cancer, leukemia or CNS tumor, according to maternal use of specific types of hormonal contraception. 2

Table S2. ATC-codes and classification of hormonal contraception............................... 3

Table S3. Perinatal characteristics of the study cohort. 4

Table S4. Characteristics of children who developed any type of cancer....................... 5

Table S5. Characteristics of children who developed leukemia...................................... 7

Table S6. Characteristics of children who developed a CNS-tumor. 9

Table S7. Type and dose of progestin in progestin-only products................................. 11

Table S1. Mortality in children who develop any type of cancer, leukemia or CNS tumor, according to maternal use of specific types of hormonal contraception.

	**Children with any type of cancers**			**Children with leukemia**			**Children with CNS-tumors**		
	Number of children	Number of deaths	HR (95% CI)*	Number of children	Number of deaths	HR (95% CI)*	Number of children	Number of deaths	HR (95% CI)*
**Any type**									
Previous use	1 864	225	1 (reference)	548	52	1 (reference)	446	84	1 (reference)
**Combined contraceptives**									
Oral contraception									
Recent use	251	36	1.17 (0.82-1.67)	76	11	1.34 (0.67-2.70)	63	8	0.65 (0.32-1.33)
During pregnancy	35	6	1.36 (0.62-2.94)	10	<5[£]	2.78 (0.97-7.97)	8	<5[£]	0.76 (0.10-5.67)
**Progestin-only contraceptives**									
Oral contraception									
During pregnancy	6	<5[£]	1.48 (0.23-9.54)	<5[£]	<5[£]	8.00 (1.44-44.39)			
Non-oral contraceptives									
Recent use	1864	7	1.51 (0.73-3.09)	8	<5[£]	1.66 (0.25-10.80)	15	<5[£]	1.52 (0.57-4.07)

Exposure categories with zero number of deaths are not shown.
During refers to use during pregnancy.
Recent use refers to use 3 months or less before pregnancy start (except for non-oral progestin only products [for further specification, see the method section]).
Previous use refers to use more than 3 months before pregnancy start (except for non-oral progestin only products [for further specification, see the method section]).
*Stratified by year of birth (categories: 1996-1999, 2000-2004, 2005-2009, 2010-2014, 2015-2018) and maternal age at birth (categories: <28, 28-31, 32-35, >35).
[£]Exact number <5 or equivalent is blinded according the interpretation of General Data Protection Regulation by Statistics Denmark.
Of note, some HRs are not shown when estimation was not possible due to few observations.
HR = Hazard ratio.

Table S2. ATC-codes and classification of hormonal contraception.

Type of hormonal contraception	ATC-code(s)
**Combined contraception**	
Oral	G03AA01, G03AA03, G03AA05, G03AA07, G03AA09-12, G03AA14, G03AA16<br>G03AB03-06, G03AB8<br>G03HB01
Non-oral	G02BB01<br>G03AA13
**Progestin-only contraception**	
Oral	G03AC01-03<br>G03AC09
Non-oral	G02BA03, G03DA02*<br>G03AC06, G03AC08
**Emergency contraception**	G03AD01, G03AD

ATC = anatomical therapeutic chemical.
*Only drugs with the trade name Depo-Provera.

Table S3. Perinatal characteristics of the study cohort.

Perinatal factors	No. (%)			
	During pregnancy	Recent use	Previous use	No use
**Number of children**	17 513 (1.2)[a]	154 381 (10.8)[b]	952 800 (66.8)[c]	301 698 (21.2)
Cesarean delivery	3 068 (17.5)	29 020 (18.8)	197 227 (20.7)	55 622 (18.4)
Missing	0 (0.0)	0 (0.0)	0 (0.0)	0 (0.0)
Birth weight (g)				
<2500	970 (5.5)	7 594 (4.9)	48 264 (5.1)	16 012 (5.3)
2500-4500	15 950 (91.1)	141 205 (91.5)	871 763 (91.5)	274 446 (91.0)
>4500	487 (2.8)	4 847 (3.1)	28 054 (2.9)	9 250 (3.1)
Missing	106 (0.6)	735 (0.5)	4 719 (0.5)	1 990 (0.7)
Median (IQR)	3490 (3110-3835)	3520 (3170-3870)	3510 (3156-3860)	3500 (3120-3850)
Gestational length (wk)				
<37	1 233 (7.0)	9 894 (6.4)	64 405 (6.8)	19 366 (6.4)
37-42	16 233 (92.7)	144 192 (93.4)	887 207 (93.1)	281 125 (93.2)
>42	47 (0.3)	295 (0.2)	1 188 (0.1)	1 207 (0.4)
Missing	0 (0.0)	0 (0.0)	0 (0.0)	0 (0.0)
Median (IQR)	40 (38-40)	40 (39-41)	40 (38-40)	40 (39-41)
Multiplicity	395 (2.3)	5 502 (3.6)	37 807 (4.0)	13 618 (4.5)
Missing	0 (0.0)	0 (0.0)	0 (0.0)	0 (0.0)
Down syndome[d]	14 (0.1)	85 (0.05)	596 (0.1)	338 (0.1)
Missing	0 (0.0)	0 (0.0)	0 (0.0)	0 (0.0)
Neurofibromatosis[e]	11 (0.1)	71 (0.05)	390 (0.04)	141 (0.05)
Missing	0 (0.0)	0 (0.0)	0 (0.0)	0 (0.0)
Other congenital disorders[f]	1 885 (10.8)	16 485 (10.7)	101 972 (10.7)	32 737 (10.8)
Missing	0 (0.0)	0 (0.0)	0 (0.0)	0 (0.0)

[a]During refers to use during pregnancy.
[b]Recent use refers to use 3 months or less before pregnancy start (except for non-oral progestin only products [for further specification, see the method section]).
[c]Previous use refers to use more than 3 months before pregnancy start (except for non-oral progestin only products [for further specification, see the method section]).
[d]ICD-10 code Q90*
[e]ICD-10 code Q85*
[f]ICD-10 code Q00-Q99* (excluding codes Q85* and Q90*).
Abbreviations: BMI = body mass index. wk = weeks. g = grams. No = number. IQR = interquartile range, ICD = International Classification of Diseases (ICD) codes version ICD-10.

Table S4. Characteristics of children who developed any type of cancer.

Characteristics	No. (%)			
	**During pregnancy**	**Recent use**	**Previous use**	**No use**
**No. of children with any type of cancer**	43 (1.4)[a]	304 (10.0)[b]	1 864 (61.6)[c]	816 (27.0)
**Child characteristics**				
Year of birth				
1996-1999	14 (32.6)	98 (32.2)	489 (26.2)	565 (69.2)
2000-2004	17 (40.5)	88 (29.0)	589 (31.6)	146 (17.9)
2005-2009	<10 (<15)[£]	70 (23.3)	451 (24.2)	76 (9.3)
2010-2014	6 (14.3)	37 (12.3)	269 (14.6)	24 (2.9)
2015-2018	<5 (<5)[£]	11 (3.7)	66 (3.6)	5 (0.6)
Median (IQR)	2002 (2000-2007)	2003 (1999-2009)	2004 (2000-2009)	1998 (1997-2002)
Sex				
Male	28 (65.1)	165 (54.3)	978 (52.5)	434 (53.2)
Female	15 (34.9)	139 (45.7)	879 (47.2)	381 (46.7)
Missing	0 (0.0)	0 (0.0)	7 (0.4)	<5 (<1)[£]
Birth order				
First	19 (44.2)	141 (46.4)	851 (46.7)	295 (36.2)
Second or higher	24 (55.8)	163 (53.6)	1 014 (54.4)	519 (63.6)
Missing	0 (0.0)	0 (0.0)	0 (0.0)	<5 (<1)[£]
**Parental characteristics**				
Origin (mother)				
Danish or descendant of immigrant[d]	<40 (<95)[£]	290 (95.4)	1 746 (94.4)	620 (76.0)
Immigrant	<5 (<10)[£]	14 (4.6)	104 (5.6)	196 (24.0)
Missing	0 (0.0)	0 (0.0)	0 (0.0)	0 (0.0)
BMI[$] (mother)				
<25	8 (19.1)	80 (26.7)	612 (32.8)	79 (9.7)
25-30	<10 (<20)[£]	47 (15.7)	227 (12.2)	29 (3.6)
31-35	<5 (<5)[£]	9 (3.0)	93 (5.0)	13 (1.6)
>35	<5 (<5)[£]	5 (1.7)	52 (2.8)	6 (0.7)
Missing	25 (58.1)	159 (53)	880 (47.2)	689 (84.4)
Maternal education[e]				
Basic	14 (32.6)	69 (22.7)	375 (20.1)	179 (21.9)
Vocational	22 (51.2)	136 (44.7)	796 (42.7)	360 (44.1)
Higher	7 (16.3)	96 (31.6)	667 (35.8)	200 (24.5)
Missing	0 (0.0)	<5 (<5)[£]	26 (1.4)	77 (9.4)
Paternal education[e]				
Basic	10 (23.3)	60 (19.7)	377 (20.2)	171 (21.0)
Vocational	22 (51.2)	170 (55.9)	903 (48.4)	362 (44.4)
Higher	6 (14.0)	68 (22.4)	531 (28.5)	211 (25.9)
Missing	5 (11.6)	6 (2)	53 (2.8)	72 (8.8)
Maternal age at birth (y)				
<28	21 (48.8)	131 (43.1)	573 (30.7)	209 (25.6)

28-31	11 (25.6)	100 (32.9)	650 (34.9)	263 (32.2)
31-35	<10 (<20)[£]	49 (16.1)	419 (22.5)	198 (24.3)
>35	<5 (<10)[£]	24 (7.9)	222 (11.9)	146 (17.9)
Missing	0 (0.0)	0 (0.0)	0 (0.0)	0 (0.0)
Median (IQR)	28 (25-32)	28 (25-31)	30 (27-33)	31 (27-34)
Paternal age at birth (y)				
<28	10 (23.3)	84 (27.6)	351 (18.8)	112 (13.7)
28-31	14 (32.6)	103 (33.8)	573 (30.7)	189 (23.2)
31-35	10 (23.3)	59 (19.4)	491 (26.3)	231 (28.3)
>35	<10 (<15)[£]	57 (18.8)	435 (23.3)	270 (33.1)
Missing	<5 (<10)[£]	<5 (<5)[£]	14 (0.8)	14 (1.7)
Median (IQR)	31 (27-33)	30 (27-34)	32 (28-35)	33 (30-37)
Maternal smoking[f]*	9 (20.9)	56 (18.4)	299 (16.0)	118 (14.5)
Missing	6 (14.0)	35 (11.5)	139 (7.5)	181 (22.2)
Maternal infertility[g]	<5 (<5)[£]	7 (2.3)	141 (7.6)	107 (13.1)
Missing	0 (0.0)	0 (0.0)	0 (0.0)	0 (0.0)

[a]During refers to use during pregnancy
[b]Recent use refers to use 3 months or less before pregnancy start or during pregnancy (except for non-oral progestin only products (for further specification, see the method section)).
[c]Previous use refers to use more than 3 months before pregnancy start (except for non-oral progestin only products (for further specification, see the method section)).
[d]Defined as having 2 parents without Danish citizenship and who were not born in Denmark.
[e]Highest attained education before birth of the child. Basic indicates mandatory school grades 9-10; vocational, secondary school and vocational education; higher, short-, medium-, and long-term higher education.
[f]Maternal smoking measured in the first trimester of pregnancy. [*]Information on maternal smoking was only available from 1998 onwards.
[g]ICD-8 code 628 and ICD-10 code N97.
[$]Information on BMI was only available from 2004 onwards.
[£]Exact number <5 or equivalent is blinded according the interpretation of General Data Protection Regulation by Statistics Denmark.
Abbreviations: BMI = body mass index. wk = weeks. No = number. IQR = interquartile range, ICD = International Classification of Diseases (ICD) codes version ICD-10.

Table S5. Characteristics of children who developed leukemia.

Characteristics	No. (%)			
	**During pregnancy**	**Recent use**	**Previous use**	**No use**
**No. of children with leukemia**	13 (1.6)[a]	86 (10.5)[b]	548 (66.6)[c]	176 (21.4)
**Child characteristics**				
Year of birth				
1996-1999	5 (38.5)	30 (34.9)	118 (21.5)	102 (58.0)
2000-2004	5 (38.5)	22 (25.6)	180 (32.9)	37 (21.0)
2005-2009	<5 (<5)[£]	22 (25.6)	141 (25.7)	31 (17.6)
2010-2014	<5 (<25)[£]	<15 (<15)[£]	89 (16.2)	6 (3.4)
2015-2018	0 (0.0)	<5 (<5)[£]	20 (3.7)	0 (0.0)
Median (IQR)	2001 (2000-2005)	2004 (1999-2008)	2005 (2001-2009)	2000 (1997-2004)
Sex				
Male	6 (46.2)	47 (54.7)	278 (50.7)	109 (61.9)
Female	7 (53.9)	39 (45.4)	268 (48.9)	67 (38.1)
Missing	0 (0.0)	0 (0.0)	<5 (<1)[£]	0 (0.0)
Birth order				
First	5 (38.5)	37 (43.0)	254 (46.4)	63 (35.8)
Second or higher	8 (61.5)	49 (57.0)	294 (53.7)	113 (64.2)
Missing	0 (0.0)	0 (0.0)	0 (0.0)	0 (0.0)
**Parental characteristics**				
Origin (mother)				
Danish or descendant of immigrant[d]	<15 (<95)[£]	83 (96.5)	514 (93.8)	128 (72.7)
Immigrant[d]	<5 (<10)[£]	<5 (<5)[£]	34 (6.2)	48 (27.3)
Missing	0 (0.0)	0 (0.0)	0 (0.0)	0 (0.0)
BMI[$] (mother)				
<25	<5 (<25)[£]	25 (29.1)	202 (36.9)	25 (14.2)
25-30	<5 (<10)[£]	12 (14.0)	69 (12.6)	10 (5.7)
31-35	<5 (<10)[£]	<10 (<10)[£]	28 (5.1)	<10 (<5)[£]
>35	0 (0)	<5 (<5)[£]	14 (2.6)	<5 (<5)[£]
Missing	8 (61.5)	42 (48.8)	235 (42.9)	132 (75.0)
Maternal education[e]				
Basic	<5 (<25)[£]	17 (19.8)	105 (19.2)	33 (18.8)
Vocational	<10 (<60)[£]	46 (53.5)	230 (43.0)	90 (51.1)
Higher	<5 (<25)[£]	22 (25.6)	206 (37.6)	39 (22.2)
Missing	0 (0.0)	<5 (<5)[£]	7 (1.3)	14 (8.0)
Paternal education[e]				
Basic	<5 (<10)[£]	10 (11.6)	104 (19.0)	29 (16.5)
Vocational	8 (61.5)	53 (61.6)	274 (50.0)	91 (51.7)
Higher	<5 (<20)[£]	21 (24.4)	157 (28.7)	38 (21.6)
Missing	<5 (<20)[£]	<5 (<5)[£]	13 (2.4)	18 (10.2)
Maternal age at birth (y)				
<28	5 (38.5)	32 (37.2)	160 (29.2)	42 (23.9)

28-31	<5 (<25)[£]	29 (33.7)	179 (32.7)	47 (26.7)
31-35	<5 (<25)[£]	20 (23.3)	136 (24.8)	38 (21.6)
>35	<5 (<20)[£]	5 (5.8)	73 (13.3)	49 (27.8)
Missing	0 (0.0)	0 (0.0)	0 (0.0)	0 (0.0)
Median (IQR)	31 (27-32)	29 (25-32)	30 (27-33)	31 (28-36)
Paternal age at birth (y)				
<28	<5 (<20)[£]	23 (26.7)	94 (17.2)	16 (9.1)
28-31	<5 (<20)[£]	28 (32.6)	162 (29.6)	33 (18.8)
31-35	<5 (<35)[£]	16 (18.6)	143 (26.1)	51 (29.0)
>35	<5 (<35)[£]	19 (22.1)	145 (26.5)	73 (41.5)
Missing	<5 (<10)[£]	0 (0)	<5 (<1)[£]	<5 (<2)[£]
Median (IQR)	33 (30-37)	30 (27-34)	32 (29-36)	34 (31-39)
Maternal smoking[f]*	<5 (<20)[£]	15 (17.4)	70 (12.8)	23 (13.1)
Missing	<5 (<25)[£]	8 (9.3)	31 (5.7)	29 (16.5)
Maternal infertility[g]	0 (0.0)	<5 (<5)[£]	47 (8.6)	37 (21.0)
Missing	0 (0.0)	0 (0.0)	0 (0.0)	0 (0.0)

[a]During refers to use during pregnancy
[b]Recent use refers to use 3 months or less before pregnancy start or during pregnancy (except for non-oral progestin only products (for further specification, see the method section)).
[c]Previous use refers to use more than 3 months before pregnancy start (except for non-oral progestin only products (for further specification, see the method section)).
[d]Defined as having 2 parents without Danish citizenship and who were not born in Denmark.
[e]Highest attained education before birth of the child. Basic indicates mandatory school grades 9-10; vocational, secondary school and vocational education; higher, short-, medium-, and long-term higher education.
[f]Maternal smoking measured in the first trimester of pregnancy. [*]Information on maternal smoking was only available from 1998 onwards.
[g]ICD-8 code 628 and ICD-10 code N97.
[$]Information on BMI was only available from 2004 onwards.
[£]Exact number <5 or equivalent is blinded according the interpretation of General Data Protection Regulation by Statistics Denmark.
Abbreviations: BMI = body mass index. wk = weeks. No = number. IQR = interquartile range, ICD = International Classification of Diseases (ICD) codes version ICD-10.

Table S6. Characteristics of children who developed a CNS-tumor.

Characteristics	No. (%)			
	**During pregnancy**	**Recent use**	**Previous use**	**No use**
**No. of children with CNS-tumors**	9 (1.2)[a]	81 (10.7)[b]	446 (58.9)[c]	221 (29.2)
**Child characteristics**				
Year of birth				
1996-1999	<5 (<25)[£]	20 (24.7)	115 (25.8)	159 (72.0)
2000-2004	6 (66.7)	23 (28.4)	153 (34.3)	38 (17.2)
2005-2009	0 (0.0)	23 (28.4)	110 (24.7)	19 (8.6)
2010-2014	0 (0.0)	<15 (<15)[£]	51 (11.4)	5 (2.3)
2015-2018	<5 (<15)[£]	<5 (<5)[£]	17 (3.8)	0 (0.0)
Median (IQR)	2004 (2002-2005)	2005 (2001-2009)	2004 (2000-2009)	1998 (1997-2001)
Sex				
Male	<10 (<80)	39 (48.2)	216 (48.4)	97 (43.9)
Female	<5 (<25)[£]	42 (51.9)	229 (51.4)	124 (56.1)
Missing	0 (0.0)	0 (0.0)	<5 (<1)[£]	0 (0.0)
Birth order				
First	<5 (<45)[£]	33 (40.7)	193 (43.3)	77 (34.8)
Second or higher	<10 (<60)[£]	48 (59.3)	253 (56.7)	142 (64.3)
Missing	0 (0.0)	0 (0.0)	0 (0.0)	0 (0.0)
**Parental characteristics**				
Origin (mother)				
Danish or descendant of immigrant[d]	<10 (<90)[£]	<80 (<100)[£]	417 (93.5)	181 (81.9)
Immigrant[d]	<5 (<15)[£]	<5 (<5)[£]	29 (6.5)	40 (18.1)
Missing	0 (0.0)	0 (0.0)	0 (0.0)	0 (0.0)
BMI[$] (mother)				
<25	<5 (<25)[£]	20 (24.7)	137 (30.8)	18 (8.1)
25-30	<5 (<25)[£]	16 (19.8)	49 (11.0)	5 (2.3)
31-35	0 (0.0)	<5 (<5)[£]	29 (6.5)	<5 (<5)[£]
>35	<5 (<15)[£]	<5 (<5)[£]	18 (4.0)	<5 (<5)[£]
Missing	<5 (<45)[£]	41 (50.6)	213 (47.8)	196 (88.7)
Maternal education[e]				
Basic	<5 (<45)[£]	22 (27.2)	103 (23.1)	51 (23.1)
Vocational	<10 (<60)[£]	37 (45.7)	166 (37.2)	100 (45.3)
Higher	0 (0.0)	20 (24.7)	170 (38.1)	53 (24.0)
Missing	0 (0.0)	<5 (<5)[£]	7 (1.6)	17 (7.7)
Paternal education[e]				
Basic	<5 (<35)[£]	18 (22.2)	84 (18.8)	55 (24.9)
Vocational	<10 (<60)[£]	49 (60.5)	225 (50.5)	95 (43.0)
Higher	0 (0.0)	12 (14.8)	121 (27.1)	50 (22.6)
Missing	<5 (<15)[£]	<5 (<5)[£]	16 (3.6)	21 (9.5)
Maternal age at birth (y)				
<28	<10 (<80)[£]	36 (44.4)	139 (31.2)	59 (26.7)

28-31	<5 (<15)[£]	28 (34.6)	163 (36.6)	75 (33.9)
31-35	0 (0.0)	12 (14.8)	91 (20.4)	51 (23.1)
>35	<5 (<15)[£]	5 (6.2)	53 (11.9)	36 (16.3)
Missing	0 (0.0)	0 (0.0)	0 (0.0)	0 (0.0)
Median (IQR)	25 (24-27)	28 (25-31)	30 (27-33)	30 (27-34)
Paternal age at birth (y)				
<28	<5 (<35)[£]	19 (23.5)	94 (21.1)	30 (13.6)
28-31	<5 (<45)[£]	31 (38.3)	143 (32.1)	64 (29.0)
31-35	0 (0.0)	14 (17.3)	115 (25.8)	55 (24.9)
>35	<5 (<15)[£]	17 (21.0)	91 (20.4)	66 (29.9)
Missing	<5 (11.1)	0 (0.0)	<5 (<5)[£]	6 (2.7)
Median (IQR)	29 (24-31)	30 (28-34)	31 (28-35)	32 (29-37)
Maternal smoking[f]*	<5 (<35)[£]	19 (23.5)	80 (17.9)	39 (17.7)
Missing	<5 (<15)[£]	9 (11.1)	27 (6.0)	53 (24.0)
Maternal infertility[g]	0 (0.0)	<5 (<5)[£]	33 (7.4)	29 (13.1)
Missing	0 (0.0)	0 (0.0)	0 (0.0)	0 (0.0)

[a]During refers to use during pregnancy

[b]Recent use refers to use 3 months or less before pregnancy start or during pregnancy (except for non-oral progestin only products (for further specification, see the method section)).

[c]Previous use refers to use more than 3 months before pregnancy start (except for non-oral progestin only products (for further specification, see the method section)).

[d]Defined as having 2 parents without Danish citizenship and who were not born in Denmark.

[e]Highest attained education before birth of the child. Basic indicates mandatory school grades 9-10; vocational, secondary school and vocational education; higher, short-, medium-, and long-term higher education.

[f]Maternal smoking measured in the first trimester of pregnancy. [*]Information on maternal smoking was only available from 1998 onwards.

[g]ICD-8 code 628 and ICD-10 code N97.

[$]Information on BMI was only available from 2004 onwards.

[£]Exact number <5 or equivalent is blinded according the interpretation of General Data Protection Regulation by Statistics Denmark.

Abbreviations: BMI = body mass index. wk = weeks. No = number. IQR = interquartile range, ICD = International Classification of Diseases (ICD) codes version ICD-10.

Table S7. Type and dose of progestin in progestin-only products.

**Hormonal contraception**	**ATC-code(s)**	**Product**	**DDD**	**U**
**Progestin-only contraception**				
Oral				
	G03AC01	Norethisterone	0.35/300	mg/mcg
	G03AC02	Lynestrenol	0.5	mg
	G03AC03	Levornorgestrel	30	mcg
	G03AC09	Desogestrel	75	mcg
Non-oral				
	G02BA03	Levonorgestrel	13.5	mg
	G03DA02	Medroxyprogesterone (Depo-Provera)	5	mg
	G03AC06	Medroxyprogesterone (Depo-Provera)	5	mg
	G03AC08	Etonogestrel	68	mcg

Abbreviations: DDD = defined daily dose, U = unit, mg = milligram, mcg = microgram.
ATC = anatomical therapeutic chemical
